# Utility of Contrast-Enhanced Transabdominal Ultrasonography to Diagnose Early Chronic Pancreatitis

**DOI:** 10.1155/2015/393124

**Published:** 2015-05-19

**Authors:** Nobuaki Azemoto, Teru Kumagi, Tomoyuki Yokota, Masashi Hirooka, Taira Kuroda, Mitsuhito Koizumi, Yoshinori Ohno, Hirofumi Yamanishi, Masanori Abe, Morikazu Onji, Yoichi Hiasa

**Affiliations:** ^1^Department of Gastroenterology and Metabology, Ehime University Graduate School of Medicine, Toon, Ehime 791-0295, Japan; ^2^Department of Community Medicine, Ehime University Graduate School of Medicine, Toon, Ehime 791-0295, Japan; ^3^Center for Liver-Biliary-Pancreatic Diseases, Matsuyama Red Cross Hospital, Matsuyama, Ehime 790-0826, Japan; ^4^Internal Medicine, Saiseikai Imabari Hospital, Imabari, Ehime 799-1502, Japan

## Abstract

*Purpose*. The purpose of this study was to establish the relationship between the grade of chronic pancreatitis (CP) and pancreatic blood flow as measured by contrast-enhanced transabdominal ultrasonography (CEUS) and to diagnose early CP easily. *Methods*. This pilot study was conducted in 8 patients with CP, 7 patients with early CP, and 6 control participants. After injecting 0.015 mL/kg of perflubutane by manual bolus, values in one region of interest (ROI) in pancreatic parenchyma and one ROI including the superior mesenteric artery (SMA) were measured. *Results*. The ratio of blood flow in the SMA and pancreatic parenchyma increased with grade of CP and was significantly higher in patients with CP (5.41; 2.10–11.02) than in patients with early CP (2.46; 1.41–5.05) and control participants (2.32; 1.25–3.04) (*P* = 0.0279, *P* = 0.0142, resp.). The ratio of blood flow in the SMA and pancreatic parenchyma correlated with grade of CP (rs = 0.5904, *P* = 0.0048). *Conclusion*. The ratio of blood flow correlates with grade of CP on CEUS. This safe and convenient method may be useful to diagnose early CP.

## 1. Introduction

Chronic pancreatitis (CP) is characterized by two distinct features: persistence of inflammation and progressive irreversible fibrosis [1]. Progression of CP leads to development of diabetes mellitus and furthermore pancreatic cancer (PC) [2]. Thus it is clinically important to make a diagnosis of CP at an early stage to prevent its progression and complications. To meet this need, the Japan Pancreas Society (JPS) proposed a new concept called “early CP” in 2009 [3]. However, endoscopic ultrasonography (EUS) and/or endoscopic retrograde cholangiopancreatography are required to diagnose early CP. Recent studies have shown that vascularity on contrast-enhanced transabdominal ultrasonography (CEUS) correlates with the pathological grade of fibrosis in patients with autoimmune pancreatitis and PC [4–7]. Previous studies have also demonstrated that CP with advanced fibrosis is associated with reduced blood vessel density and decreased pancreatic blood flow [8–10]. Several studies have correlated the grade of CP (degree of fibrosis) with decreased pancreatic blood flow on dynamic contrast-enhanced magnetic resonance imaging (MRI) [11, 12]. However, the utility of CEUS in identifying degree of fibrosis in CP, especially early CP, remains unclear.

We speculated that CEUS using perflubutane might play a key role in classifying the grade of CP. The purpose of this study was to establish the relationship between the grade of CP and pancreatic blood flow as measured by CEUS.

## 2. Materials and Methods

This pilot study was conducted with a total of 21 participants at Ehime University Hospital. We classified three categories of CP, early CP, and control according to the criteria of CP proposed by the JPS. The JPS defined that the criteria comprise six items: (i) characteristic imaging findings, (ii) characteristic histological findings, (iii) repeated upper abdominal pain, (iv) abnormal pancreatic enzyme levels in the serum or urine, (v) abnormal pancreatic exocrine function, and (vi) continuous heavy drinking of alcohol equivalent to or more than 80 g/day of pure ethanol. Definite and probable findings are set for items (i) and (ii), and the standards are specified for (iv) and (v). Patients with more than two items among (iii), (iv), (v), and (vi) who show EUS or endoscopic retrograde cholangiopancreatography (ERCP) findings of early CP as well are diagnosed with early CP [3]. Twenty-one patients were comprised of 8 patients with CP, 7 patients with early CP, and 6 controls without pancreatic diseases, diabetes mellitus, and alcohol dependency. CP or early CP was diagnosed according to the criteria proposed by the JPS as confirmed by existing results from US, EUS, computed tomography (CT) or MRI, and laboratory tests [3]. Informed consent was obtained from each participant, and the study protocol conformed to the ethical guidelines of the 1975 Declaration of Helsinki. The study protocol was approved by the Local Ethics Committee at the Ehime University Graduate School of Medicine (Approval ID number 1108003).

**Figure 1:**
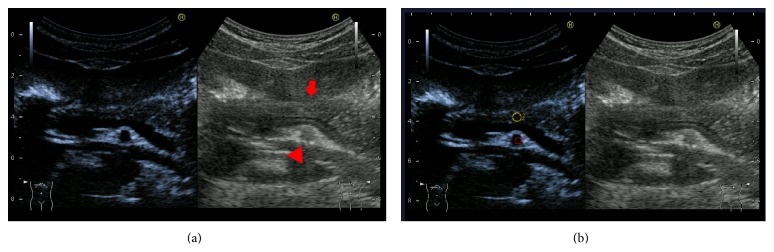
(a) Pancreatic parenchyma (arrow) is visualized together with the superior mesenteric artery (SMA, arrowhead). (b) We defined one region of interest (ROI) in the pancreatic parenchyma and one ROI including the SMA.

**Figure 2:**
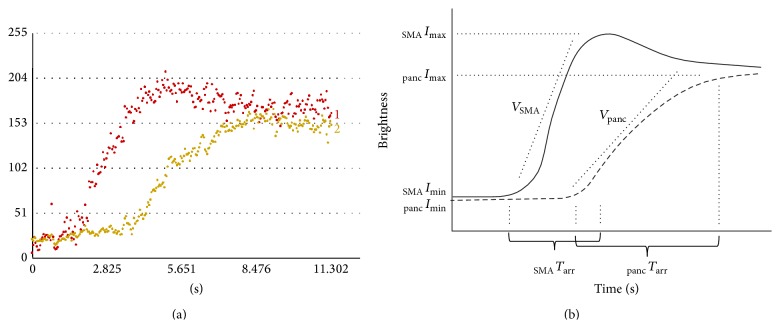
(a) Actual presentation of the time-intensity curve (TIC). (b) Schematic presentation of TIC. *I*_max⁡_, maximum intensity of brightness; *I*_min⁡_, minimum intensity of brightness; *T*_arr_, time to arrival at *I*_max⁡_; *V*, tilt to peak intensity.

CEUS was performed by one operator (N.A.), a board-certified fellow of the Japan Society of Ultrasonics in Medicine, using a Preirus (Hitachi, Tokyo, Japan) and a 3.5 MHz convex probe in all patients. The protocol for CEUS examinations was based on previous reports [6, 13, 14]. First, it was mandatory that the pancreas was visualized together with the superior mesenteric artery (SMA) (Figure 1(a)). Second, 0.015 mL/kg body weight of perflubutane was injected as a manual bolus, followed by a flush of 5.0 mL of normal saline solution using a 22 G needle into an antecubital or cubital vein. CEUS was conducted in harmonic imaging mode with a low mechanical index (0.23) to avoid bubble disruption. Third, we defined one region of interest (ROI) in pancreatic parenchyma and one ROI including the SMA after the examination. ROIs were selected within the visible area of the pancreatic body. The area of the ROIs ranged from 0.11 to 0.15 cm^2^ (Figure 1(b)). Based on the data obtained, maximum and minimum intensity of brightness (*I*_max⁡_ and *I*_min⁡_), time to arrival at *I*_max⁡_ (*T*_arr_), and tilt to peak intensity (*V* = (*I*_max⁡_ − *I*_min⁡_)/*T*_arr_) were derived (Figures 2(a) and 2(b)). We next calculated the ratio of *V* for the SMA (*V*_SMA_) to *V* for the pancreatic parenchyma (*V*_panc_) as *V*_SMA_/*V*_panc_, and differences between CP, early CP, and control groups were compared. We then examined correlations between several factors and *V*_SMA_/*V*_panc_. We also evaluated the utility of using *V*_SMA_/*V*_panc_ to diagnose early CP.

Data collected also included body mass index (BMI), body surface area, pancreatic volume/body surface area (PV/BSA), serum amylase, serum lipase, LDL-cholesterol, triglyceride, serum C-peptide immunoreactivity (CPR) levels, HbA1c (NGSP), and pancreatic function diagnostant (PFD) test.

All statistical analyses were performed using JMP for Windows version 8 software (SAS International, Cary, NC). Continuous variables were expressed as median and ranges. Differences between two groups were analyzed using Wilcoxon rank-sum test. Differences among three groups were analyzed using the Kruskal-Wallis test. Categorical data were analyzed using *χ* ^2^ test. Correlations between grade of CP and *V*_SMA_/*V*_panc_ and between *V*_SMA_/*V*_panc_ and various variables were analyzed using nonparametric correlation coefficients (Spearman's *ρ*). Two-tailed significance was defined in all analyses as a value of *P* < 0.05.

## 3. Results

### 3.1. Clinical Characteristics of Participants

Clinical characteristics of participants with CP or early CP and controls were shown in Table 1. No significant differences in age, sex, BMI, or pancreatic volume/body surface area were apparent among participants with CP, early CP, and controls. Serum levels of amylase, lipase, LDL-cholesterol, triglyceride, CPR, and HbA1c also were not significant. But PFD test was significantly different among three groups (0.0474).

### 3.2. Comparison of _panc_ *I*_max⁡_, _panc_ *T*_arr_, and *V*_panc_ according to Grade of CP

*I*_max⁡_ for the pancreatic parenchyma (_panc_ *I*_max⁡_), *T*_arr_ for the pancreatic parenchyma (_panc_ *T*_arr_), and *V*_panc_ were compared among three groups (Table 2). No significant differences in _panc_ *I*_max⁡_, _panc_ *T*_arr_, and *V*_panc_ were shown.

### 3.3. Comparison of *V*_SMA_/*V*_panc_ according to Grade of CP

**Figure 3:**
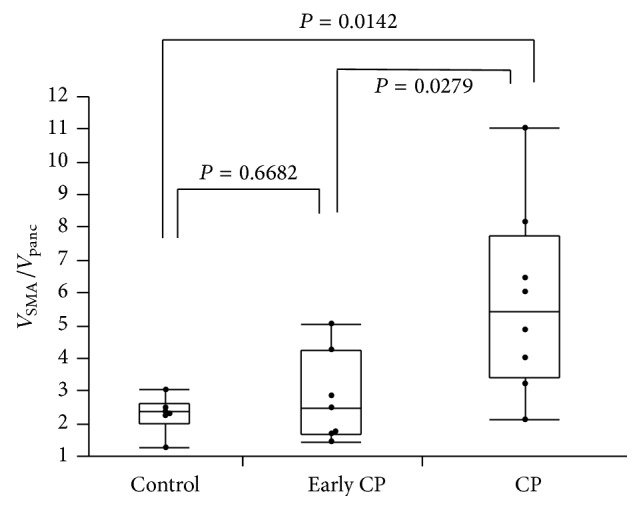
The value of *V*_SMA_/*V*_panc_ shows the ratio of *V* for the SMA to *V* for pancreatic parenchyma and increases according to the grade of CP. Box contains values between 25th and 75th percentiles of the value of *V*_SMA_/*V*_panc_ (central line, median). Vertical lines represent minimum and maximum. Values were significantly higher in patients with CP than in patients with early CP and controls (*P* = 0.0279 and *P* = 0.0142, resp.). The value of *V*_SMA_/*V*_panc_ also correlated with the grade of CP (Spearman's *ρ*: rs = 0.5904, *P* = 0.0048).

Values of *V*_SMA_/*V*_panc_ increased according to the grade of CP and were significantly different among three groups (*P* = 0.0209; Table 3). *V*_SMA_/*V*_panc_ of CP were significantly higher than those of early CP and control (*P* = 0.0279, *P* = 0.0142, resp.). Although values of *V*_SMA_/*V*_panc_ did not differ between early CP and control (*P* = 0.6682), values of *V*_SMA_/*V*_panc_ correlated with the grade of CP (Spearman's *ρ*: rs = 0.5904, *P* = 0.0048; Figure 3).

### 3.4. Correlation of  *V*_SMA_/*V*_panc_ and Various Variables

PFD test was the only significant clinical characteristic factor among three groups (Table 1). We investigated the relationship between value of *V*_SMA_/*V*_panc_ and PFD test. However, there was no correlation between two factors (Table 4). We also examined the relationship between various variables and value of *V*_SMA_/*V*_panc_ (Table 4), so that PV/BSA and HbA1c were correlated with value of *V*_SMA_/*V*_panc_ (PV/BSA: rs = 0.7027, *P* = 0.0008, HbA1c: rs = 0.4244, *P* = 0.0134; Table 4).

## 4. Discussion

We showed the ratio of blood flow observed by CEUS correlated with progression of CP (Figure 3). Schilling et al. reported that blood flow of the pancreas was significantly diminished in patients with CP [9]. Similarly, a histological study by Angelis et al. found that fibrosis of the pancreas and changes in blood vessels were observed in 80–100% and 14–44% of patients with CP, respectively [8]. However, these studies were conducted among patients with CP showing advanced fibrosis and did not include cases of CP with minimal fibrosis, namely, early CP. Whether these observations are reproducible for early CP and whether decreased blood flow as well as progression of fibrosis is occurring simultaneously thus remain unclear. The concept of “early CP” was recently proposed by the JPS, in a revision of the diagnostic criteria for CP [3]. Early CP is now defined as a prestage of CP. We demonstrated that blood flow in the pancreas decreases in a stepwise manner concordant with these criteria, and CEUS may thus offer a useful method to classify the degree of CP.

Second, we were indeed able to classify grade of CP by CEUS into groups of CP, early CP, or control (Figure 3). We demonstrated that *V*_SMA_/*V*_panc_ values increased according to the grade of CP. Values of *V*_SMA_/*V*_panc_ were significantly higher in patients with CP than in patients with early CP and controls (*P* = 0.0279 and *P* = 0.0142, resp.; Figure 3). Furthermore, we showed that *V*_SMA_/*V*_panc_ correlated with pancreatic volume/body surface area and HbA1c, although no significant correlation was between *V*_SMA_/*V*_panc_ and PFD test. Serum levels of amylase, lipase, LDL-cholesterol, triglyceride, and CPR also were not significant (Table 4). We speculated that endocrine dysfunction of pancreas gradually decreases according to reduction in pancreatic volume, but exocrine dysfunction decreases suddenly. In this region, values of *V*_SMA_/*V*_panc_ were correlated with HbA1c but were not correlated with PFD test.

We demonstrated that CEUS using perflubutane had the potential to allow diagnosis of early CP. Although perflubutane is a frequently used agent, mainly in the diagnosis of liver tumors [13], two reports have shown the utility of this contrast agent in the diagnosis of PC [6] and intraductal papillary mucinous neoplasm [15]. Matsubara et al. also recently showed the utility of perflubutane in the diagnosis of PC using endoscopic ultrasound [14]. However, CEUS using perflubutane had never been studied in terms of the diagnosis of CP or early CP. CP is a strong risk factor for PC, and the prognosis of PC is extremely poor [2]. Detection of PC as early as possible is thus desirable. However, no strategies to satisfy this need have yet been devised. In the near feature, screening program may be built because enclosure of the high risk group of PC from an early stage is enabled according to diagnosis of early CP using CEUS.

Several limitations of this study must be kept in mind when interpreting the results. First, the number of participants was small. This may be why we were unable to show any difference in *V*_SMA_/*V*_panc_ between patients with early CP and CP. We should conduct a multicenter study. Second, this method is not applicable for obese individuals, since observing both the pancreas and SMA simultaneously is frequently difficult in such individuals. In contrast, the advantages of this method were simplicity, safety, and convenience.

## 5. Conclusion

The ratio of blood flow on CEUS correlated with grade of CP. This method is safe and convenient. However, larger studies should be conducted in the future to confirm this potential.

## Figures and Tables

**Table 1:** Clinical characteristics of participants.

	Control (*N* = 6)	Early chronic pancreatitis (*N* = 7)	Chronic pancreatitis (*N* = 8)	*P*
Age	61.5 (28–74)	66 (54–69)	58.5 (40–67)	0.0913^*^
Sex (M : F)	5 : 1	5 : 2	8 : 0	0.2826^**^
BMI (kg/m^2^)	21.4 (18.5–24.5)	21.2 (19.7–29.6)	20.8 (16.2–26.1)	0.9036^*^
Body surface area (m^2^)	1.56 (1.47–1.92) (*N* = 4)	1.64 (1.44–1.70) (*N* = 7)	1.62 (1.51–1.79) (*N* = 8)	0.6105^*^
PV/BSA (cm^2^/m^2^)	38.83 (36.67–42.56) (*N* = 3)	27.26 (18.94–46.38) (*N* = 7)	19.23 (7.48–38.42) (*N* = 8)	0.0879^*^
Amylase (U/L)	100 (38–170)	105 (58–544)	66 (33–127)	0.1675^*^
Lipase (U/L)	33 (27–52) (*N* = 5)	48 (32–132) (*N* = 7)	21 (10–121) (*N* = 8)	0.0564^*^
LDL-cholesterol (mg/dL)	125 (56–155)	92 (60–99)	65 (40–127)	0.0901^*^
Triglyceride (mg/dL)	74 (41–206)	122 (54–399)	72 (39–177)	0.2596^*^
Serum CPR (ng/mL)	1.28 (0.47–2.53)	2.02 (0.17–2.74)	1.55 (0.23–3.17)	0.4047^*^
HbA1c (%: NGSP)	5.7 (5.3–6.0) (*N* = 5)	5.9 (4.7–6.5) (*N* = 7)	8.5 (5.4–9.4) (*N* = 8)	0.0581^*^
PFD test (%)	72.9 (69.6–75.4) (*N* = 5)	71.6 (60.2–78.0) (*N* = 7)	56.3 (18.7–72.7) (*N* = 8)	0.0474^*^

^*^Kruskal-Wallis test, ^**^ *χ* ^2^ test.

BMI: body mass index, BSA: body surface area, and PV: pancreatic volume.

CPR: C-peptide immunoreactivity and PFD: pancreatic function diagnostant.

**Table 2:** Comparison of _panc_ *I*_max⁡_, _panc_ *T*_arr_, and *V*_panc_ according to grade of chronic pancreatitis.

	Control (*N* = 6)	Early chronic pancreatitis (*N* = 7)	Chronic pancreatitis (*N* = 8)	*P* ^*^
_panc_ *I*_max⁡_ (brightness)	113.8 (23.4–177.2)	94.8 (61.7–193.3)	111.2 (51.1–144.7)	0.7207
_panc_ *T*_arr_ (sec)	4.82 (3.02–7.20)	4.90 (2.58–7.45)	5.15 (3.33–6.87)	0.6876
*V*_panc_ (brightness/sec)	22.28 (4.04–33.58)	11.40 (3.54–35.19)	10.27 (5.18–19.64)	0.4760

^*^Kruskal-Wallis test.

_panc_ *I*_max⁡_: *I*_max⁡_ for the pancreatic parenchyma and *I*_max⁡_ and *I*_min⁡_: maximum and minimum intensity of brightness.

_panc_ *T*_arr_: time to arrival at *I*_max⁡_ of the pancreatic parenchyma and *T*_arr_: time to arrival at *I*_max⁡_.

*V*_panc_: *V* for the pancreatic parenchyma, *V* = (*I*_max⁡_ − *I*_min⁡_)/*T*_arr_.

**Table 3:** Comparison of *V*_SMA_/*V*_panc_ according to grade of chronic pancreatitis.

	Control (*N* = 6)	Early chronic pancreatitis (*N* = 7)	Chronic pancreatitis (*N* = 8)	*P* ^*^
*V*_SMA_/*V*_panc_	2.32 (1.25–3.04)	2.46 (1.41–5.05)	5.41 (2.10–11.02)	0.0209

^*^Kruskal-Wallis test.

*V*_SMA_: *V* for the superior mesenteric artery and *V*_panc_: *V* for the pancreatic parenchyma.

**Table 4:** Relationship between *V*_SMA_/*V*_panc_ and various variables.

	rs	*P*
Age	0.2146	0.0913^*^
BMI (kg/m^2^)	0.0713	0.9822^*^
Body surface area (m^2^)	0.0695	0.8866^*^
PV/BSA (cm^2^/m^2^)	0.7027	0.0008^*^
Amylase (U/L)	0.2316	0.1037^*^
Lipase (U/L)	0.2841	0.2288^*^
LDL-cholesterol (mg/dL)	0.1568	0.3331^*^
Triglyceride (mg/dL)	0.1612	0.8165^*^
Serum CPR (ng/mL)	0.0796	0.9688^*^
HbA1c (%: NGSP)	0.4244	0.0134^*^
PFD test (%)	0.3619	0.2156^*^

^*^Spearman's *ρ*.

BMI: body mass index, BSA: body surface area, and PV: pancreatic volume.

CPR: C-peptide immunoreactivity and PFD: pancreatic function diagnostant.
